# P300-Based Brain-Computer Interface Speller: Usability Evaluation of Three Speller Sizes by Severely Motor-Disabled Patients

**DOI:** 10.3389/fnhum.2020.583358

**Published:** 2020-10-29

**Authors:** M. Teresa Medina-Juliá, Álvaro Fernández-Rodríguez, Francisco Velasco-Álvarez, Ricardo Ron-Angevin

**Affiliations:** Departamento de Tecnología Electrónica, Universidad de Málaga, Malaga, Spain

**Keywords:** amyotrophic lateral sclerosis (ALS), patient, brain-computer interface (BCI), electroencephalography (EEG), P300, speller, size, usability

## Abstract

Brain-computer interface (BCI) spellers allow severe motor-disabled patients to communicate using their brain activity without muscular mobility. Different visual configurations of the widely studied P300-based BCI speller had been assessed with healthy and motor-disabled users. However, the speller size (in terms of cm) had only been assessed for healthy subjects. We think that the speller size might be limiting for some severely motor-disabled patients with restricted head and eye movements. The usability of three speller sizes was assessed for seven patients diagnosed with amyotrophic lateral sclerosis (ALS) and a participant diagnosed with Duchenne muscular dystrophy (DMD). This is the first usability evaluation of speller size with severely motor-disabled participants. Effectiveness (in the online results) and efficiency (in the workload test) of the medium speller was remarkably better. Satisfaction was significantly the highest with the medium size speller and the lowest with the small size. These results correlate with previously described findings in healthy subjects. In conclusion, the speller size should be considered when designing a speller paradigm, especially for motor-disabled individuals, since it might affect their performance and user experience while controlling a BCI speller.

## Introduction

Amyotrophic lateral sclerosis (ALS) is a neurological disorder that degenerates the upper and lower motor neurons, leading to paralysis and eventually death (Patterson and Grabois, 1986). However, other functions such as sensory perception or intellectual abilities are usually preserved. ALS patients may gradually enter a locked-in state (LIS), where they are only able to slightly move their eyes and make other small residual movements (Bauer et al., 1979; Murguialday et al., 2011). On the other hand, Duchenne muscular dystrophy (DMD) is a genetic progressive muscular degeneration disorder which also leads to paralysis and eventually death (Emery et al., 2015). As with ALS patients, DMD patients usually preserve their sensory perception and intellectual abilities (Emery et al., 2015). Some of the main differences between ALS and DMD disorders are that DMD is genetic, usually starts at early ages—childhood—and often evolves slowly; while ALS cause is unknown, usually starts at later ages—adulthood—and normally evolves faster than DMD.

Researchers in the field of assistive technology have developed different systems to these patients with an alternative communication channel, e.g., eye-tracker (Pal et al., 2017) or brain-computer interface (BCI) systems (Birbaumer, 2006). The latter allows people to interact with their environment using brain activity without any peripheral nerve involvement [see Nicolas-Alonso and Gomez-Gil (2012) for an extended review of BCI]. As these patients may not be able to control gaze at some stages of their condition, they may require a BCI to establish communication to provide them with some autonomy in their daily life.

According to Nicolas-Alonso and Gomez-Gil (2012), BCI systems most often use electroencephalography (EEG) to measure a subject’s brain activity to study different waveforms. This article will focus on the P300 evoked potentials, which are positive peaks appearing 300 ms after an odd stimulus happens. This signal is typically used by P300-based BCI systems named virtual spellers (Rezeika et al., 2018). The first oddball paradigm was proposed by Farwell and Donchin (1988); it had a matrix of letters and numbers, with each matrix’s column and row flashing pseudo-randomly. The subject must pay attention to a particular character while the rows and columns flash, and when his/her target character is flashed, the P300 potential is evoked and recorded by the BCI system to determine which letter the user wants to select.

Numerous visual factors, e.g., colors or the nature of the stimulus, have been assessed in a P300 speller (Ikegami et al., 2012; Acqualagna et al., 2013; Li et al., 2015). However, the speller size has barely been tested. Sellers et al. (2006) compared two matrixes with different numbers of elements (3 × 3 and 6 × 6) and dimensions (5.44°H × 7.07°W and 8.30°H × 10.90°W of visual angle, respectively). In that study, the small matrix showed better accuracy; however, it is unknown if this difference is due to the visual angle defined by the speller or the number of elements in the speller. On the other hand, Salvaris and Sepulveda (2009) tested three visual configurations of the speller relative to the background color, the distance between symbols, and symbol size. However, the symbol size and symbol distance parameters were not varied together to find the optimal actual speller dimensions. Nonetheless, their results showed that the matrix with the smallest size gave the worst performance for both conditions. Li et al. (2011) compared three screen sizes (computer monitor: 17″, 1,200 × 1,000 pixels; GPS: 9″, 700 × 500 pixels; cell phone: 5″, 260 × 425 pixels) and concluded that better performance was achieved with the largest screen size. However, no information about the speller and symbol size was provided. Therefore, the differences between resolutions and the lack of the exact measurements of the spellers and symbols prevent satisfactory conclusions about the symbol size. Finally, Ron-Angevin et al. (2019) assessed three speller sizes—under overt and covert attention—using the usability approach (ISO, 2000). They found that the medium speller size (9.98 × 9.98 cm; 9.5°H × 9.5°W) was the most convenient since it offered high effectiveness, efficiency and satisfaction.

It is important to highlight that none of the quoted articles used motor-disabled participants. Therefore, it is necessary to verify these results with potential end-users. Ron-Angevin et al. (2019) studied three speller sizes with healthy subjects under overt attention conditions. Nevertheless, this condition might not be representative of motor-disabled patients as most of them may only preserve the residual head and eye movements at some stages of their disease (Patterson and Grabois, 1986; Emery et al., 2015). In this sense, an adequate speller size has to be established considering the limitations of patients’ gaze and head movement. While large sizes might be hard to handle and tiring due to the required muscular movements, a too-small speller could be less tiring but lead to inaccuracy in the perception of the speller’s elements.

Hence, the present study aims to assess the effect of three different speller sizes, in terms of the delimited visual angle, to determine the most appropriate speller size for severely motor-disabled participants. The sizes studied were proposed by Ron-Angevin et al. (2019). Moreover, a usability approach (ISO, 2000) was employed for the evaluation with three factors studied: effectiveness, efficiency, and satisfaction.

## Materials and Methods

### Participants

Seven Spanish participants diagnosed with ALS (P1-P7, all males, aged 64.43 ± 11.1) and one diagnosed with DMD (P8, male, aged 26) volunteered for the study. Two ALS volunteers (P9 and P10) could not take part in the experiment because the signal classifier was unable to generate usable weights for their brain waveform classification matrix, so they were unable to control the system. Every participant, or the corresponding legal representative, provided written informed consent.

According to self-reports, the participants had no history of neurological or psychiatric illness besides ALS or DMD and had normal or corrected to normal vision (Table 1). The patients were referred by the ALS Association of Andalusia, and none of them had prior experience with BCI systems. The test took place in their home but was coordinated by the research group UMA-BCI^1^. The study was approved by the Ethics Committee of the University of Malaga and met the ethical standards of the Helsinki Declaration.

**Table 1:** Participants’ information.

Patient	Age (years)	Years post-diagnosis	ALSFRS-R (Cedarbaum et al., 1999)	Regular communication channel	Alternative technologies
P1	64	4	12	Voice	None
P2	73	5	29	Hands	Eye-tracker, voice-synthesizer
P3	56	4	0	Gaze, blinks	Eye-tracker
P4	65	20	19	Voice	Facial-recognition, voice-recognition
P5	45	3	54	Voice	None
P6	70	3	10	Voice, gaze	Eye-tracker
P7	78	7	29	Handwriting	None
P8	26	18	31	Voice	Eye-tracker, assistive keyboard

*Note: ALSFRS-R stands for Revised ALS Functional Rating Scale*.

### EEG Recording and Signal Processing

EEG data were registered using an acti-Champ amplifier (Brain Products GmbH, Munich, Germany) and recorded using the electrode positions: Fz, Cz, Pz, Oz, P3, P4, PO7, and PO8 according to the 10/20 international system. The electrodes were referenced in TP8 and grounded in AFz. A band-pass filter at 0.1–30 Hz was applied, and the Notch filter (50 Hz) was on. BCI2000 (Schalk et al., 2004) was used to control all aspects of EEG data collection and processing except for the analysis of the waveforms, which was carried out with MATLAB’s toolbox EEGLAB (Delorme and Makeig, 2004).

### Spelling Paradigms

**Figure 1:**
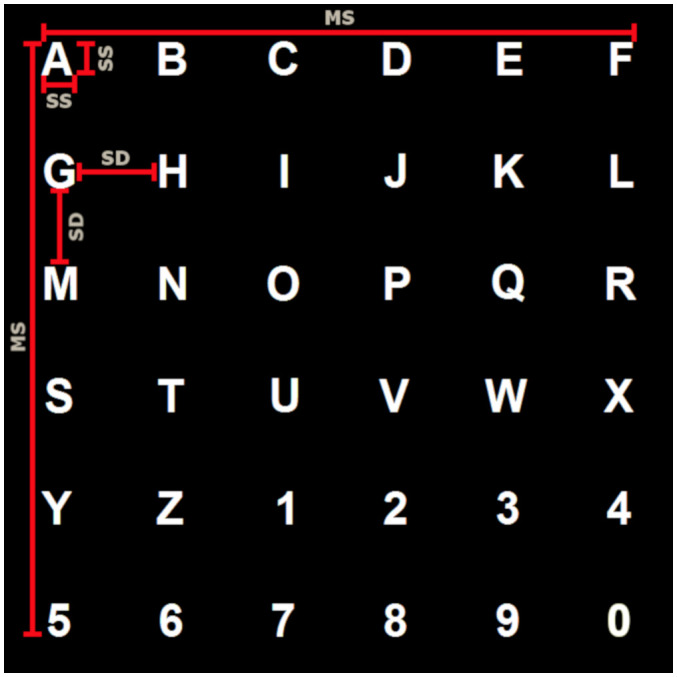
Speller’s size parameters. MS stands for “speller size,” SS for “symbol size,” and SD for “symbol distance.”

Three speller sizes were designed according to Ron-Angevin et al. (2019), where the three of them had a similar appearance as the classic P300 speller: characters in gray color (stimulus off) were presented over a black background; when the “flash” occurred (i.e., stimulus on), the characters turned to white color. A flash lasted 128 ms and the time between flashes (inter-stimuli interval, ISI) was 128 ms as well. After every set of flashes, there was a pause of 6 s except for patients P1, P2, and the first speller of P3 who used 2 s due to a mistake while applying the experimental protocol. This timing difference might not have been a problem as discussed below in the Discussion section. Each sequence of stimulation consisted of flashing one time every row and column (which implies that each character flashed two times per sequence). During the calibration and online phase, ten sequences were used. The spellers consisted of a 6 × 6 character matrix with the English alphabet and numbers from 0 to 9 (Figure 1).

According to Ron-Angevin et al. (2019), the used symbol sizes and distance between columns and rows were selected as follows (Table 2):

(1) The largest size was the one proposed by Treder and Blankertz (2010), which is usually used by other researchers like Brunner et al. (2010) and Brunner et al. (2011). This matrix size defined a visual angle of 13.96° both horizontally and vertically. The symbol size delimited a visual angle of 1.12°H × 1.12°W (H and W stand for height and width, respectively), and the separation between characters was 1.46° horizontally and vertically.
(2) In the opposite case, the smallest size was selected following what (Salvaris and Sepulveda, 2009) reported as the minimum symbol size that could be used without loss of performance. In this case, the delimited visual angle by each symbol was 0.4°H × 0.45°W. In the present study, the selected symbol size keeps the same metrics, defining a square visual angle of 0.4°H × 0.4°W. The separation between characters was calculated proportionally to the size and separation in the large size case: 0.5° horizontally and vertically.
(3) The selected medium size was the middle size between the large and small ones. The visual angle defined by the matrix was 9.5°H × 9.5°W, the one defined by the symbols was 0.75°H × 0.75°W, and the angle defined by the vertical and horizontal separation was 1° for each of them.

**Table 2:** Values of the spellers’ size parameters.

Size	Measures (cm)			Visual angle (°)
	Speller size	Symbol size	Symbol distance	Speller size
Small	5.27	0.42	0.55	5°H × 5°W
Medium	9.98	0.79	1.04	9.5°H × 9.5°W
Large	14.69	1.17	1.53	14°H × 14°W

Note: MS stands for “speller size,” SS for “symbol size,” and SD for “symbol distance.”

### Procedure

The experimental protocol consisted of three sessions of 60 ± 10 min. The order of the spellers’ usage was counterbalanced between participants. The time between sessions was in a range of 5 h and three days. Each session consisted of three phases: (i) a calibration phase; (ii) an online spelling phase; and, finally, (iii) subjective questionnaires fill out phase.

#### Calibration Task

Participants were asked to mentally count the times that the first desired letter flashed and, when the first set of flashes was over, to focus on the next letter. They had to repeat this procedure until the word was completed. The Spanish words to calibrate were “LUNA,” “RAMO,” “KILO,” and “2015.” Before each word calibration started, the participants were reminded of the word to spell. Only the last three calibrated words were used to obtain the speller classifier’s weights by applying a stepwise linear discriminant analysis (SWLDA) to offer the corresponding feedback during the online phase.

#### Online Task

Three Spanish words were spelled one after the other: “CHAT,” “PURE,” and “1935.” If the classifier selected a wrong letter, participants had no option to correct the mistake. Participants were reminded of what words to spell during the test. This time, each typed letter was represented in a text box placed above the matrix.

#### Subjective Questionnaires

The last part of each session consisted of answering three different questionnaires: two visual analog scales (VAS) questionnaires and the NASA-TLX test (Hart and Staveland, 1988). Finally, when the three sessions were concluded, a comparative questionnaire for the three sizes was filled out.

### Usability Evaluation

The evaluation of the usability was carried out considering the approach proposed by ISO (2000), including three measures: effectiveness, efficiency, and satisfaction.

#### Effectiveness

Effectiveness was related to the degree of correctness with which the user completed the tasks. For this purpose, different results were obtained:

(i) *Accuracy* during the classification phase, which indicates the classifier accuracy after it analyzed and classified the EEG data of a participant in each sequence.
(ii) *Error performance* (*EP*) in the online phase, which was calculated by dividing the number of wrong selections by the total of selections and multiplied by 100; and percentage of participants that met the *MEP30* criterion, which correlates to the 30% threshold that Kübler et al. (2001) indicated as the maximum *EP* allowed to establish an efficient communication system.
(iii) Analyses of the ERP target and no-target waveforms and the *amplitude difference* (*AD*) of the ERP stimuli waveforms (i.e., ERP target waveform—ERP non-target waveform).

#### Efficiency

The efficiency relates to the resources expended to complete a task. In this case, three results were considered:

(i) Subjective workload assessed using *NASA-TLX*, which evaluated the mental, physical, and temporal demand, as well as the performance, effort, and frustration perceived by the participant.
(ii) *VAS fatigue* (Kim et al., 2010), whose weight varied from 0 to 10 (where 0 is the minimum and 10 the maximum), was used to evaluate the level of fatigue experienced during the test.
(iii) The second VAS of questions regarding the speller’s perception was applied to evaluate the difficulty in perceiving the characters (*Q1*), the difficulty in perceiving the characters away from the center (*Q2*), and the difficulty in distinguishing the different rows and columns (*Q3*).

#### Satisfaction

Finally, satisfaction was related to the users’ attitude. The subjective feelings about the different speller sizes were analyzed using the comparative questionnaire based on the System Usability Scale (Brooke, 1996). This questionnaire compared complexity, stressfulness, controllability, tiredness, comfortableness, and user preference for the spellers. Specifically, participants had to assign the spellers the ranks “the least,” “the intermediate,” and “the most” preferred.

A *satisfaction index* was calculated as in Ron-Angevin et al. (2019) to provide a general perspective of this questionnaire. Firstly, the satisfaction’s related variables were categorized as positive (*controllable*, *comfortable*, and *preferred*) or negative (*complex*, *stressful*, and *tiring*). Finally, each rank was associated with a score: rank 1 (the least) as ±1, rank 2 (the intermediate) as ±2, and rank 3 (the most) as ±3. The sign of the score depended on the category of the variables.

### Statistical Analyses

The present study employed factorial analyses—unifactorial, since only the speller size factor was studied—with three levels (one for each speller size). Specifically, an ANOVA or a Friedman’s test was applied depending on whether the sample met, respectively, the assumption of normality or not (*accuracy*, *EP*, all variables relative to the efficiency dimension and the *satisfaction index*). Likewise, for the ANOVA, the Greenhouse-Geisser correction was applied in case the sphericity assumption was not satisfied. Afterward, for the multiple comparison analysis, the Bonferroni’s correction method was used. On the other hand, for those variables that aimed to study whether the distribution in each of the variables depended on the speller size, a Fisher’s exact test was employed (concretely, *MEP30* threshold, and the variables related to the System Usability Scale). The EEGLAB software (Delorme and Makeig, 2004) was used to carry out the ANOVA related to the study of the speller size factor on the ERP waveform.

## Results

The collected results from the patients are presented according to the usability criteria.

### Effectiveness

#### Classification Accuracy During the Calibration Phase

**Figure 2:**
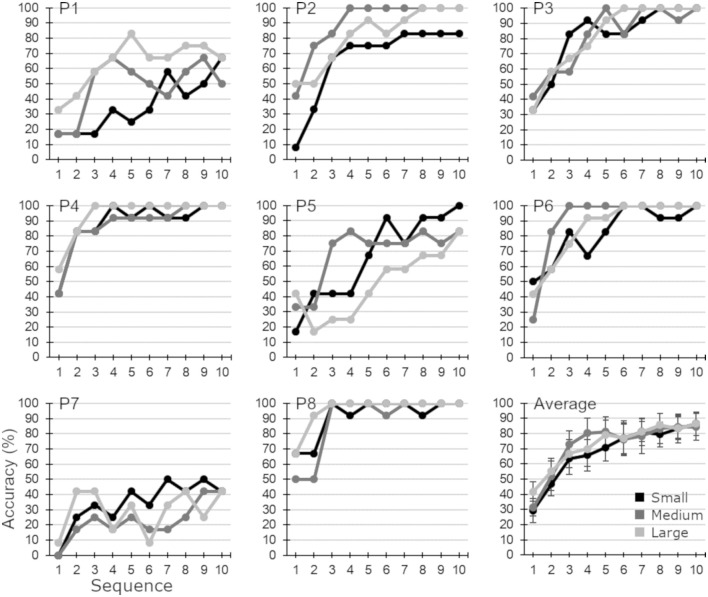
Accuracy (%) obtained by every participant and average (±SD) in each condition and sequence.

According to Friedman’s tests, no significant differences in accuracy between sizes were found in any sequence (Figure 2).

#### Error Performance During Online Spelling

The ANOVA relative to the *EP* for the three speller sizes did not show significant differences between conditions (Table 3). The percentage of participants that achieved the *MEP30* threshold was 50% (four participants out of eight) for small size and 62.5% (five participants out of eight) for medium and large sizes, so no significant difference was noted according to the Fisher’s exact test.

**Table 3:** Error performance (%) of every participant in each condition.

Participant	Size		
	Small	Medium	Large
P1	83.33	41.67	61.11
P2	33.33	25	25
P3	0	0	16.67
P4	8.33	8.33	0
P5	25	41.67	58.33
P6	33.33	8.33	16.67
P7	66.67	75	83.33
P8	0	0	0
Average (±SD)	31.25 ± 30.46	25 ± 26.35	32.29 ± 30.67

#### Event-Related Potentials During the Calibration Phase

**Figure 3:**
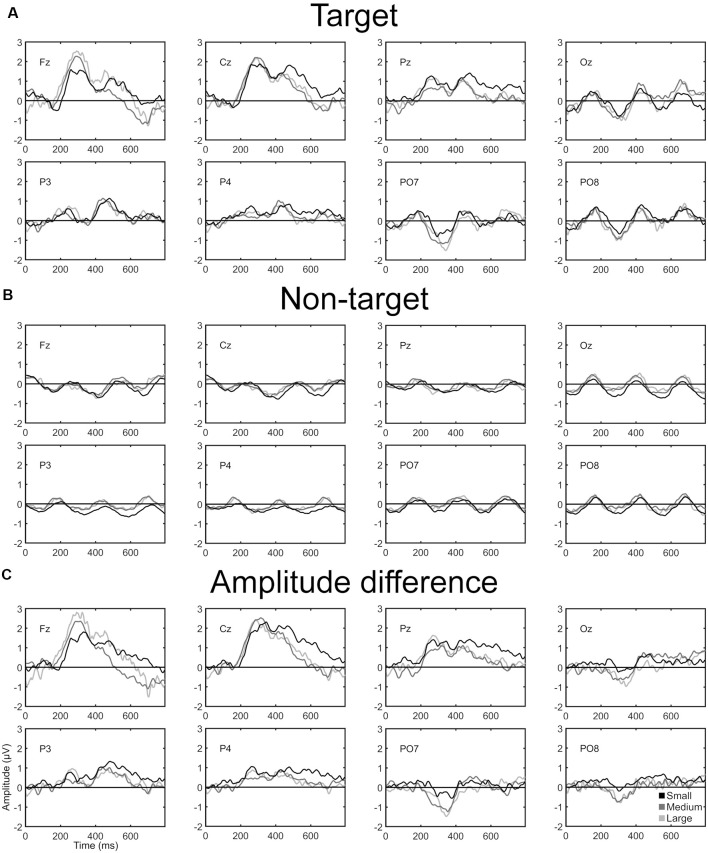
**(A)** ERP target grand average, **(B)** ERP non-target grand average, and **(C)** amplitude difference (AD; i.e., ERP target waveform − ERP non-target waveform). Every graph is presented over time (ms) and shows the grand average of each channel and speller.

Figure 3 shows the ERP waveform of target and non-target stimulus, and the difference between them (*AD*) for each condition and channel. Significant differences were not found in any time interval between conditions.

### Efficiency

The results obtained from the subjective questionnaires are presented.

#### VAS Fatigue and NASA-TLX

According to the ANOVA, the following parameters relative to the *NASA-TLX* test offered a significant main effect produced by the speller size factor (Table 4): *physical demand* (*F*_(2,14)_ = 4.029; *p* = 0.041), *temporal demand* (*F*_(2,14)_ = 4.927; *p* = 0.024) and *effort* (*F*_(2,14)_ = 5.107; *p* = 0.022). Nevertheless, due to the multiple comparisons’ correction applied, the *post hoc* analyses only showed significant differences for the *effort* factor between the medium and small sizes (*p* = 0.012).

**Table 4:** *VAS fatigue* and *NASA-TLX* scores (mean ± standard deviation).

Parameter	Size		
	Small	Medium	Large
VAS fatigue	3.63 ± 2.5	2.63 ± 2.26	3.63 ± 2.83
Mental demand	36.88 ± 22.03	25 ± 17.73	28.13 ± 22.19
**Physical demand**	**38.75 ± 26.42**	**24.38 ± 18.21**	**24.38 ± 23.21**
**Temporal demand**	**34.38 ± 16.13**	**16.25 ± 9.91**	**33.75 ± 19.78**
Performance	35.63 ± 17	34.38 ± 18.21	25 ± 24.2
**Effort**	**48.75 ± 24.46**	**29.38 ± 20.78**	**33.75 ± 31.93**
Frustration	23.13 ± 23.75	18.13 ± 17.31	21.25 ± 30.79
Total workload	40.92 ± 15.28	29.63 ± 10.35	33.92 ± 18.7

*Note: Factors with significant differences (*p* < 0.05) are presented in bold*.

#### Perception of Subjective Questionnaires

Friedman’s test did not show significant differences in any statement, that is, the main effect produced by the speller size factor was not observed (Table 5).

**Table 5:** Scores of each perception parameter.

Parameter	Size		
	Small	Medium	Large
Q1	2.13 ± 2.8	0.75 ± 1.75	1.88 ± 2.64
Q2	2.25 ± 2.66	1.13 ± 1.73	1.75 ± 2.96
Q3	2.5 ± 2.33	1.75 ± 1.91	0.75 ± 0.89

Note: Q1 stands for “the difficulty in perceiving the characters,” Q2 for “the difficulty in perceiving the characters away from the center,” and Q3 for “the difficulty in distinguishing the different rows and columns.”

### Satisfaction

**Figure 4:**
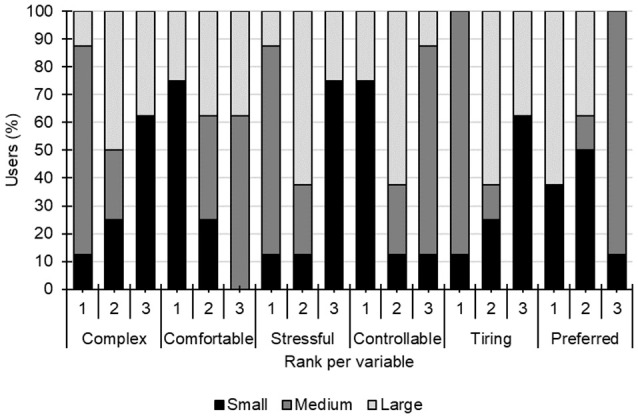
Percentage of patients that chose a Rank regarding factors for each speller. Rank 1 stands for “the least,” Rank 2 “the intermediate,” and Rank 3 “the most.”

Figure 4 shows the percentage of patients that recorded satisfaction with each speller size for each factor. According to the Fisher’s exact test, statistical differences were detected between speller sizes and percentage of participants that selected each rank in all factors: *complex* (*p* = 0.025), *comfortable* (*p* = 0.011), *stressful* and *controllable* (*p* = 0.004), tiring (*p* = 0.001), and finally, *preferred* (*p* = 0.001).

**Figure 5:**
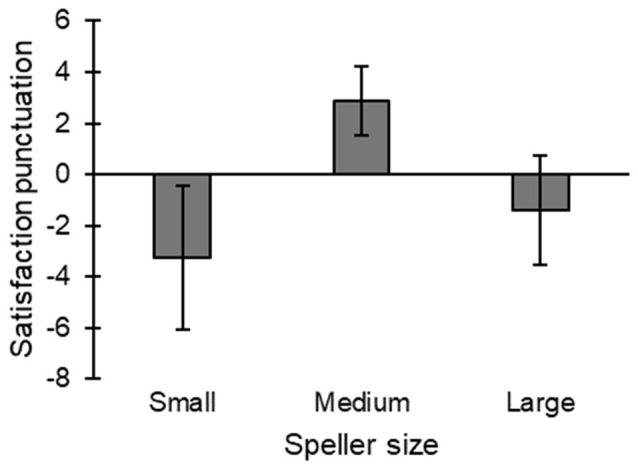
Representation of the satisfaction index regarding each speller.

Figure 5 shows the representation of the *satisfaction index*, with the medium size speller having the best and the small size the worst. The factor *speller size* showed a significant main effect (*χ*^2^_(2)_ = 9.25; *p* = 0.01). Specifically, the multiple comparison analyses showed significant differences between the medium and the small size (*p* = 0.018), and medium and large size (*p* = 0.037). However, there were no significant differences between small and large sizes.

## Discussion

In this section, the results obtained will be discussed and contextualized concerning previous articles. Specifically, to compare the results of our participants with those obtained by subjects without motor disabilities, the previous work of Ron-Angevin et al. (2019)—which is closely related to the present study—will be used.

### Effectiveness

No significant differences were found for any speller in the *accuracy* achieved in the calibration task. As shown in Figure 2, there are no clear tendencies. Likewise, considering the results obtained in the online task (Table 3) and the *MEP30* threshold, no significant differences were found within the spellers. Almost the same number of participants achieved the criterion with the three spellers: for large and medium, five out of eight (62.5%); and for small, four out of eight (50%). These results suggest that the 2-s pause between selections used in the experiments of participants P1, P2, and P3 might have not affected their results, as some participants with a 6-s pause performed even worse than the three of them. Furthermore, the patients’ scores in this study were similar to results of other studies when a speller based on the one of Farwell and Donchin (1988) and with a larger or similar sample size (e.g., Nijboer et al., 2008) and McCane et al. (2014) was used. First, in Nijboer et al. (2008), four out of six participants (i.e., 67%) reached the *MEP30* threshold; and in McCane et al. (2014), 17 out of 25 participants (i.e., 68%) overcome the same threshold; in contrast to the present study in which 62.5% participants did reach it with the large and medium sizes.

Nevertheless, a tendency is remarkable in the *EP* average results obtained in the online phase (Table 6); the lowest and best values are obtained with the medium size as patients can overcome the *MEP30* threshold only with this size. A similar tendency was found in the covert condition (Ron-Angevin et al., 2019; Table 6). Most probably, the present article could not statistically confirm this tendency due to the small sample size. The *EP* averages of the present study and the results of Ron-Angevin et al. (2019) for covert and overt attention (Table 6) indicate that the average results of the patients are substantially worse compared to those of the non-disabled individuals under overt attention, but are closer to healthy subjects under covert attention. These results might suggest that, overall, patients had greater difficulty with speller control, given their possible restricted ocular mobility. Nevertheless, when subtracting the results of those patients who reached the *MEP30* criterion for at most one speller (i.e., P1, P5, and P7), the averages obtained are considerably lower (small: 15 ± 17.08%, medium: 8.33 ± 10.21%, large: 11.67 ± 11.18%), reaching the *MEP30* criterion all spellers. The worsening of the *EP* might have been caused by, for example, these three participants’ difficulty to gaze control; however, it is not possible to verify as we do not have this information.

**Table 6:** Error performance (%) averages from the online task.

Study	Size		
	Small	Medium	Large
Present study	31.3 ± 30.5	25 ± 26.3	32.3 ± 30.7
Ron-Angevin et al. (2019) for covert attention	45.83 ± 6.7	43.75 ± 7.2	58.33 ± 7.8
Ron-Angevin et al. (2019) for overt attention	2.8 ± 1.6	4.9 ± 2.8	16.0 ± 4.5

On the other hand, it is worth noting that the performance appears to have no link to the ALSFRS-R score. As declared previously by McCane et al. (2014), this lack of relationship may be due to the ineffectiveness of the ALSFRS-R to measure the ocular deterioration of patients, which is one of the essential requirements to control a visual speller. Specifically, patient P9–who could not control the interface—obtained a score of 0 in the ALSFRS-R and had enormous difficulty in keeping his eyes open during the test. Otherwise, patient P3 had the same ALSFRS-R score, but he achieved a lower *EP* (0%, 0%, 16.7% for small, medium, and large, respectively) even compared to the average non-disabled participants of Ron-Angevin et al. (2019) for the overt condition (2.8 ± 1.6%, 4.9 ± 2.8%, 16.0 ± 4.5% for small, medium and large, respectively). Thus, some information about their ocular control should be specified.

Regarding the ERP waveforms (i.e., ERP target and non-target stimulus signals) analysis, there were no significant differences between the spellers in any time interval of any channel. Similar results were obtained by Ron-Angevin et al. (2019) with healthy subjects under the covert and overt attention paradigms, as this study did not show significant differences in amplitude nor latency regarding the speller size factor. Therefore, the expected results were obtained in the present study.

Figure 3, in the target and non-target ERP signals of the three spellers, shows a sine wave in every channel possibly due to the constant flashing of the interface, as it has a period close to the SOA (i.e., 256 ms). To remove this side effect, the AD between ERP stimulus signals was calculated. This last study did not show statistical differences. A possible P300 component is shown in Fz, Cz, and Pz between 200 and 600 ms with a maximum peak amplitude at around 300 ms. However, this component is affected by a negative peak at 400 ms that could be provoked by the sinusoidal wave. The P300 component observed in both conditions of Ron-Angevin et al. (2019) is shown in every channel and has a longer latency (between 200 and 500 ms with a maximum peak at around 400 ms) than in the present study. However, our results coincide with what declared other studies with patients (McCane et al., 2015). In the occipital zone (PO7, PO8, and Oz), a possible N200 component can be observed from 200 to 400 ms, which might have canceled the P300 component. On the other hand, this negative component was also found in Ron-Angevin et al. (2019) in the parietal-occipital zone, but only under the overt condition. Therefore, it could be inferred that the patients might possess adequate eye mobility, at least to the point of being able to fix their attention on the desired stimuli, as N200 is the earliest component that correlates with visual awareness (Railo et al., 2011).

Considering the ERP waveforms (Figure 3), the results from the calibration phase could be explained as both measures correlate, especially looking at the *AD* waveform. We think that the *AD* waveform—instead of the target or non-target ERP waveforms—might be the most interesting to analyze because it shows how different in amplitude are the ERP target and no-target signals, and thus the ease of distinguishing between both signals for the classifier. On average, no significant differences were observed between the three speller sizes in the *AD* signal nor in the classification accuracy. Specifically, participants yield the *MEP30* threshold in the 6^th^ sequence and from that sequence, the performance of the three spellers is quite similar with only small differences.

### Efficiency

Three dimensions had significant differences in the NASA-TLX results (i.e., *physical demand*, *temporal demand*, and *effort*). However, due to the applied multiple comparisons’ correction, only the medium size speller required less *effort* than the small size with a significant difference. Remarkably, the small size had the highest score in these three factors, and the medium size had the lowest in the *temporal demand* and *effort* dimensions. Interestingly, the results of the healthy subjects of Ron-Angevin et al. (2019) did not present statistical differences between spellers in the overt attention condition for any dimension, what might indicate that they were not highly affected by the speller size in terms of *total workload* while controlling a speller BCI in contrast to the motor disabled participants. Nevertheless, the average *total workload* declared by them (i.e., 40.4 ± 7.2, 38.22 ± 4.8, 41.2 ± 6.4, for the small, medium, and large sizes, respectively) is notably higher in contrast to patients (i.e., 40.92 ± 15.28, 29.63 ± 10.35, 33.92 ± 18.7, for the small, medium and large sizes, respectively). A possible explanation for these results could be that patients were more positive or optimistic during the test than the healthy participants due to their condition. Furthermore, these results suggest that: (i) the small speller size was the most complicated for the patients; and (ii) the medium size was the less demanding for patients and healthy subjects. In contrast, the average total workload of the three spellers from the present work was also smaller than described by Pasqualotto et al. (2015), whose motor-impaired participants had an average *total workload* of 47.64 ± 14.87. This difference may be explained by the lower ALSF-R in patients of Pasqualotto et al. (2015) than in our study (i.e., 15.5 ± 13.26 and 23 ± 16.61, respectively).

### Satisfaction

The medium size was selected as the best option for every dimension and the small size is the one with the worst results for most of the dimensions (i.e., for six out of seven dimensions) according to satisfaction questionnaires. On the other hand, the non-preference for the small speller could be explained by the difficulty in perceiving the different stimuli in general (i.e., *Q1*, *Q2*, and *Q3*). The results of the healthy subjects under the overt condition of Ron-Angevin et al. (2019) did not show any trend regarding the most convenient speller size since the large and medium sizes obtained similar scores. However, they showed that the small size is the worst option in the four dimensions that presented significant differences. Thus, it could be affirmed that the small size is the least convenient for patients and healthy subjects.

In the satisfaction index (Figure 5), the medium speller is the only size that had the most positive scores (significantly better than the other two sizes). Similar results were also found by Ron-Angevin et al. (2019) as the medium size was the only speller that got positive scores in both conditions (i.e., cover and overt). Therefore, it seems clear that the most convenient speller size is the medium one.

### Limitations

BCI-based studies that include results of severely motor-disabled patients usually share the limitation of having a small sample size due to the difficulty in finding patients that would like to volunteer. The present study was able to include a similar or larger sample size than one reported in the literature (Kaufmann et al., 2013; Severens et al., 2014; Speier et al., 2017; Zhang et al., 2017). Despite the limited sample size used in this article, some conclusions can be drawn from the results. On the one hand, a remarkable tendency was observed of the medium size as the one with the best *EP* results from the online phase. On the other hand, from the subjective measures, the medium size can be concluded as the most convenient size and the small size the least convenient in a significant manner. Most probably, if the sample size were larger, the trend observed in the objective measures could have been statistically affirmed and the control of different variables that may influence the system performance would have been included.

## Conclusions

This work is the first study related to speller sizes for motor disabled people. It has shown that the size of the speller matters and should be considered for this population. Furthermore, it has been proved that the most commonly used speller size (i.e., the large one) might not be the most suitable for patients.

Summarizing, in the present study the medium size is the most and the small size the least usable in terms of satisfaction dimension. Furthermore, a tendency is remarkable in the *EP* averages (from the effectiveness dimension), which highlights the medium size as the only speller that enables efficient communication according to the *MEP30* criterion. Finally, while the medium speller was selected as the least *temporal demanding* and the one that required less *effort* to control, the small size was selected as the most *physically demanding* and the one that required more *effort* according to the NASA-TLX scores (from the efficiency dimension).

The results from the objective measures show a large variability which suggests that optimization for each individual might be worthwhile. For example, P1 and P6 performed better with the medium size, P4 with the large size, P5 with the small, while P8 achieved 0% *EP* with the three spellers. On the other hand, considering the *EP* average results of the online phase and the subjective measures, it can be concluded that, among the three sizes studied, the medium size is the most convenient. Similarly, the small size can be concluded as the least convenient. Nevertheless, the optimal size should be further studied in future works knowing that it might be placed between the large and medium sizes for most patients. It should be noted that even if the optimal speller size is found, most probably in some cases the speller size will have to be adapted to the necessities of the patient.

Most probably, if the present study had a larger sample size, the medium speller could have been statistically affirmed in every usability dimension as the most suitable size. Nevertheless, this tendency has been already validated by Ron-Angevin et al. (2019) with healthy subjects.

Finally, it will be interesting to investigate other applications in the future, e.g., web-browsing or games, with the medium speller size because this size would leave more space, in contrast to the most frequently used large size, within the monitor screen for these types of applications.

## Data Availability Statement

The raw data supporting the conclusions of this article will be made available by the authors, without undue reservation.

## Ethics Statement

The studies involving human participants were reviewed and approved by Comité Ético de Experimentación de la Universidad de Málaga (CEUMA). CEUMA registry number: 51-2019-H. The patients/participants provided their written informed consent to participate in this study. Written informed consent was obtained from the individual(s) for the publication of any potentially identifiable images or data included in this article.

## Author Contributions

MM-J, ÁF-R, FV-Á, and RR-A contributed to the conception and design of the study. MM-J and FV-Á contacted the participants. MM-J, ÁF-R, and FV-Á performed the experiments. ÁF-R performed the statistical analysis. MM-J and ÁF-R wrote the first draft of the manuscript. RR-A was in charge of the funding acquisition, project administration, and supervision. All authors contributed to the article and approved the submitted version.

## Conflict of Interest

The authors declare that the research was conducted in the absence of any commercial or financial relationships that could be construed as a potential conflict of interest.

## Footnotes

^1^http://umabci.uma.es
